# Deep multiview learning to identify imaging-driven subtypes in mild cognitive impairment

**DOI:** 10.1186/s12859-022-04946-x

**Published:** 2022-09-29

**Authors:** Yixue Feng, Mansu Kim, Xiaohui Yao, Kefei Liu, Qi Long, Li Shen, for the Alzheimer’s Disease Neuroimaging Initiative

**Affiliations:** 1 Imaging Genetics Center, Stevens Institute for Neuroimaging and Informatics, Keck School of Medicine, University of South California, Los Angeles, USA; 2 Perelman School of Medicine, University of Pennsylvania, Philadelphia, USA

**Keywords:** Deep learning, Multiview learning, Multimodal imaging, Image-driven subtypes

## Abstract

**Background:**

In Alzheimer’s Diseases (AD) research, multimodal imaging analysis can unveil complementary information from multiple imaging modalities and further our understanding of the disease. One application is to discover disease subtypes using unsupervised clustering. However, existing clustering methods are often applied to input features directly, and could suffer from the curse of dimensionality with high-dimensional multimodal data. The purpose of our study is to identify multimodal imaging-driven subtypes in Mild Cognitive Impairment (MCI) participants using a multiview learning framework based on Deep Generalized Canonical Correlation Analysis (DGCCA), to learn shared latent representation with low dimensions from 3 neuroimaging modalities.

**Results:**

DGCCA applies non-linear transformation to input views using neural networks and is able to learn correlated embeddings with low dimensions that capture more variance than its linear counterpart, generalized CCA (GCCA). We designed experiments to compare DGCCA embeddings with single modality features and GCCA embeddings by generating 2 subtypes from each feature set using unsupervised clustering. In our validation studies, we found that amyloid PET imaging has the most discriminative features compared with structural MRI and FDG PET which DGCCA learns from but not GCCA. DGCCA subtypes show differential measures in 5 cognitive assessments, 6 brain volume measures, and conversion to AD patterns. In addition, DGCCA MCI subtypes confirmed AD genetic markers with strong signals that existing late MCI group did not identify.

**Conclusion:**

Overall, DGCCA is able to learn effective low dimensional embeddings from multimodal data by learning non-linear projections. MCI subtypes generated from DGCCA embeddings are different from existing early and late MCI groups and show most similarity with those identified by amyloid PET features. In our validation studies, DGCCA subtypes show distinct patterns in cognitive measures, brain volumes, and are able to identify AD genetic markers. These findings indicate the promise of the imaging-driven subtypes and their power in revealing disease structures beyond early and late stage MCI.

**Supplementary Information:**

The online version contains supplementary material available at 10.1186/s12859-022-04946-x.

## Background

Multimodal neuroimaging data are able to provide different but complementary information about brain functions that a single modality cannot [1]. One application in studying Alzheimer’s Disease (AD) is classifying cases from controls using multimodal imaging data (e.g., structural magnetic resonance imaging (MRI) and amyloid positron emission tomography (PET)) [2]. Although classification can help with effective diagnosis of AD or MCI, another application is to identify disease subtypes to assist with targeted treatment, and there have been increasing efforts to identify subtypes using multimodal data [3–6], using neuroimaging, biomarker or clinical measurements.

One method to extract subtypes is unsupervised clustering [5, 7]. However, clustering is often applied to the original features directly and fails to escape the “curse of dimensionality” [8] as we move to a high dimensional feature space-as is the case with multimodal imaging data if we were to use naive concatenation. There have been studies that proposed data fusion methods to fuse information from multimodal data and reduce dimensionality, such as multi-kernel support vector machine (SVM) [2], multimodal random forest [9], and deep learning [10, 11], but are often used for supervised tasks such as classification. The work by [12] proposed coupled nonnegative matrix factorization (C-NMF) to discover AD phenotypes and is jointly optimized with existing healthy control (HC), mild cognitive impairment (MCI) and AD groups.

To address these limitations, we proposed an unsupervised deep multiview learning framework based on canonical correlation analysis (CCA) in our previous work [13]. While traditional CCA [14] learns linear combinations of the variables in two input data views that maximize their correlation, Generalized CCA (GCCA) [15] extends CCA by learning from more than 2 views of data, and Deep CCA [16] can apply non-linear transformations using deep neural networks. Deep generalized CCA (DGCCA) [17] combine both GCCA and DCCA to learn maximally correlated components from more than 2 views. Using features learned from DGCCA, we conducted cluster analysis to identify population structure (case control groups), and genetic association analysis on candidate AD risk SNPs [18]. DGCCA shows promising results in capturing variation of multimodal data in few latent components using non-linear transformation and identifying population structure.

Patients with Mild Cognitive Impairment (MCI) show decline in cognitive functions and are at higher risk of converting to AD. To further uncover disease subtypes, we expand upon our previous work and apply the multiview learning framework on multimodal imaging data of MCI patients to facilitate early detection of AD.

To validate the imaging-driven MCI subtypes identified by clusters generated from multiview features, we compare them with those generated from single modality features. We further conducted survival analysis to investigate subtype-specific conversion to AD and genetic association. There have been studies conducting survival analysis to examine AD progression using various cognitive and imaging features [19, 20]. In our study, we used the Cox proportional hazards regression model to visualize conversion curves for the identified subtypes using baseline cognitive and brain volume measures. Further genetic association analyses performed on comparing the healthy group and each identified subtype were able to confirm existing AD risk genes (e.g. APOE, TOMM40) and discover additional genetic markers.

## Results

### Multiview learning

**Fig. 1:**
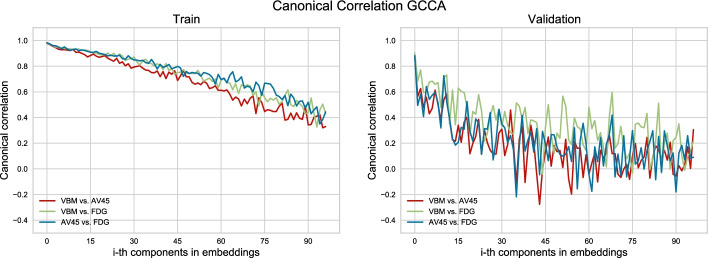
Canonical Correlation for components extracted from GCCA‘

**Fig. 2:**
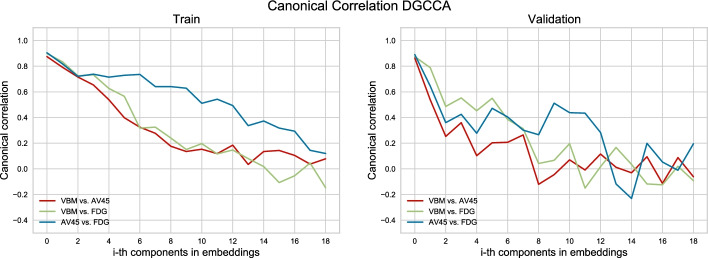
Canonical Correlation for components extracted from DGCCA

After selecting the top 94 features, explaining 68.66% variance from GCCA embeddings, and the top 20 features explaining 68.85% variance from DGCCA embeddings, we calculated the canonical correlation between modalities $corr(O_{j_1}U_{j_1}, O_{j_2}U_{j_2})$ where $j_i\in \left[ 1,2,3\right]$ corresponds to each input view, for each embedded feature from the training and validation set, see Figs. 1, 2. While the correlation between GCCA features for the training set is consistently high, all above 0.3, the validation set correlation drops after the first 15 features. For DGCCA features, although the correlation drops after the first 8 features in the training set, the validation set correlation is comparable to that of the training set. With a closer look at the modality specific correlation, both methods produce features with lower correlation between AV45 and VBM. GCCA shows higher VBM and FDG correlation in the validation set, and DGCCA shows higher AV45 and FDG correlation in the training set.

**Fig. 3:**
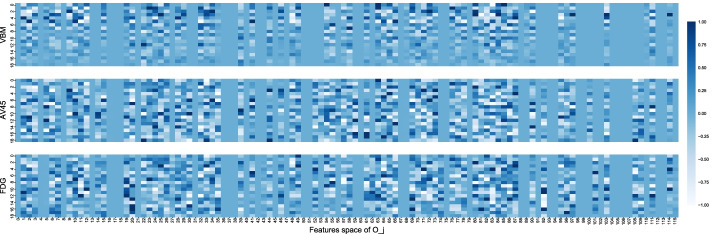
Latent features learned by DGCCA projected to each imaging modality $U_i$. The x-axis marks 116 imaging features (output of the neural network) and the y-axis marks 20 latent features

Figure 3 plots the view-dependent projection matrices learned by DGCCA ($U_i$), where the x-axis dimension is the feature space of $O_j$ (output of neural networks, $q_j=116$) and the y-axis is the embedded feature space ($k=20$). Projection for AV45 selected 83 non-zero features from the $O_j$ feature space, where that for VBM selected 74, and that for FDG selected 80. In addition, 64 features are jointly selected by all three modalities. While we show that DGCCA can learn shared information from all 3 imaging modalities, it can learn unique information as well. For instance, we can see that features for AV45 and FDG are more salient compared to VBM features especially after the first 10 embedded features. AV45 assigns heavy weighting to the feature at index 59 where the FDG zeros it out.

### Generating subtypes using clustering

The existing classification of participants into early MCI and late MCI are often characterized by the extent of cognitive decline and performance on cognitive assessments. The purpose of our study is not particularly to find subtypes that improve upon existing MCI groups, but to see if subtypes generated from imaging data can group them differently, and potentially reveal a new disease structure that can help us further understand AD progression. Since we generated subtypes using multiview learning methods from multimodal data, we compared with single modality data in evaluation to see if the GCCA or DGCCA features could be effectively learned from multimodal data.

Cluster evaluation metrics are shown in Table 1. The first step in evaluation was to see if these subtypes were indeed good clusters. Using traditional intrinsic measures, Calinski-Harabasz (CH) and Silhouette score, Exp 2 using AV45 features produced the best defined cluster, and all single modality experiments (Exp 1–3) outperformed both multiview methods. GCCA generates almost indistinguishable clusters whereas DGCCA, while not outperforming single modalities in these regards, yielded a comparable result, showing that it could produce valid clusters.

**Table 1:** Cluster evaluation

	CH	Silhouette	AMI
Exp 1 VBM	182.839	0.308	0.008
Exp 2 AV45	322.853	0.431	0.020
Exp 3 FDG	144.537	0.251	0.028
Exp 4 GCCA	2.908	0.038	-0.001
Exp 5 DGCCA	133.704	0.303	0.039

Clusters are evaluated using Calinski and Harabasz (CH) and the silhouette score as internal measures. Higher CH score and Silhouette close to 1 indicates more dense and well separated clusters, where lower CH score and Silhouette closer to 0 indicates more poorly defined and overlapping clusters. In addition, Adjusted Mutual Information (AMI) are calculated for each cluster assignment against the original EMCI/LMCI diagnosis. AMI closer to 1 means two sets of clusters are more similar where AMI close to 0 means they are more independent from each other

**Fig. 4:**
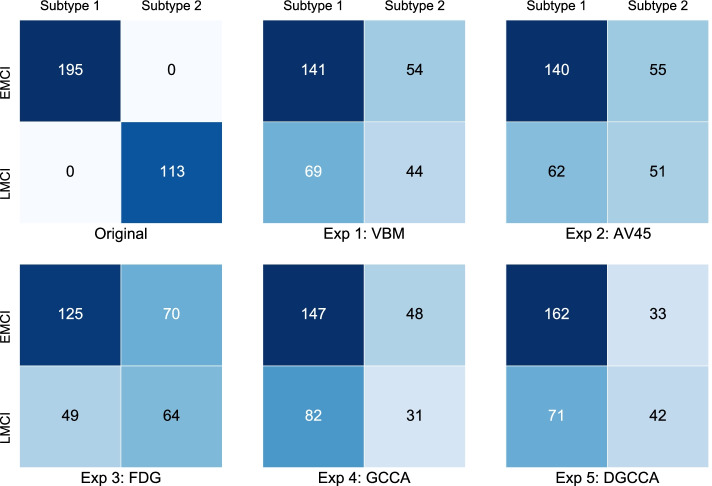
Confusion matrices of cluster assignments for each experiment

In addition to intrinsic cluster evaluation, we also plotted confusion matrices for subtypes generated from 5 experiments, shown in Fig. 4, where Subtype 1 is assigned to the cluster with the higher EMCI to LMCI ratio. For all experiments, the majority of EMCI participants are assigned to Subtype 1, where LMCI participants are more evenly distributed between the two. Only Exp 5 using DGCCA embeddings assigned more LMCI participants to Subtype 2. We also computed AMI score between the generated clusters and the EMCI/LMCI groups, where a score of 1 means that two clusters are identical and a score of 0 or negative means they are independent. Subtypes from Exp 5 using DGCCA features are the most similar to the original MCI groups out of all experiments, but a low value indicates that they are still very different.

**Fig. 5:**
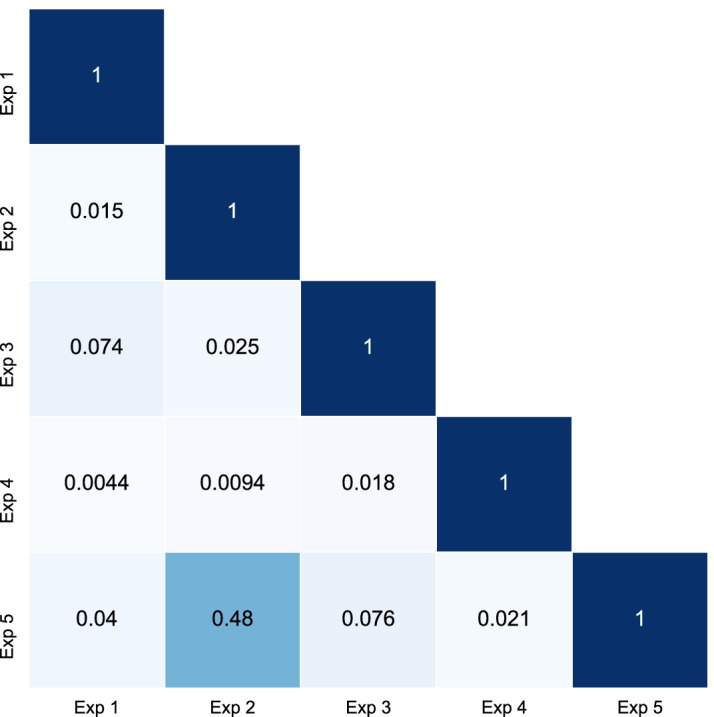
Similarity of cluster assignment between experiments

To further investigate the subtypes generated from each experiment, we computed the AMI score between each pair of experiments, shown in Fig. 5. Subtypes generated from DGCCA features are most similar to those from AV45 features. Given that Exp 2 also produced the best defined clusters, AV45 features are more discriminative. DGCCA subtypes are therefore more influenced by AV45 even though all input views to DGCCA are weighted the same.

**Fig. 6:**
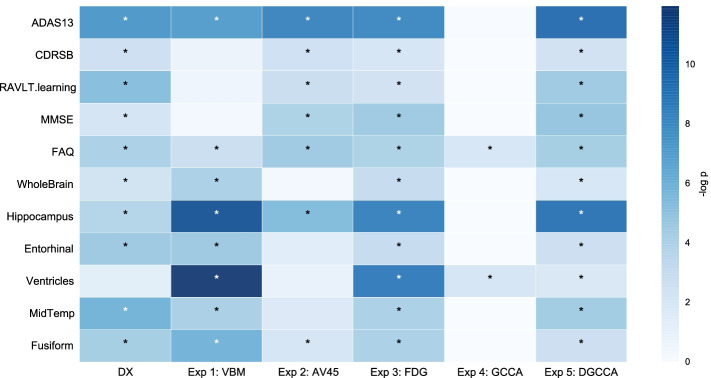
Cognitive and biomarker measurement analysis. Heatmap of -log(p) (Bonferroni-corrected at $p=0.01$) of the rank-sum test. Significant values are marked by * in cell

In addition to cluster evaluation, we also conducted Wilcoxon rank-sum test on 11 cognitive and brain volume baselines measures, and plotted the $-log_{10}(p)$ value in Fig. 6. Subtypes from FDG (Exp 3) and DGCCA (Exp 5) features show differential measure in all biomarkers. It’s worth noting that VBM and FDG show very strong signal in terms of ventricles volume and AV45 does not. While both GCCA and DGCCA produced significant result for ventricles volume and the original MCI groups (DX) does not, DGCCA shows very weak signal, which might be because DGCCA is influenced more by AV45 features. All three modalities show stronger signal in hippocampus volume and ADAS13 score, which is picked up by DGCCA features as well.

### Survival analysis

**Fig. 7:**
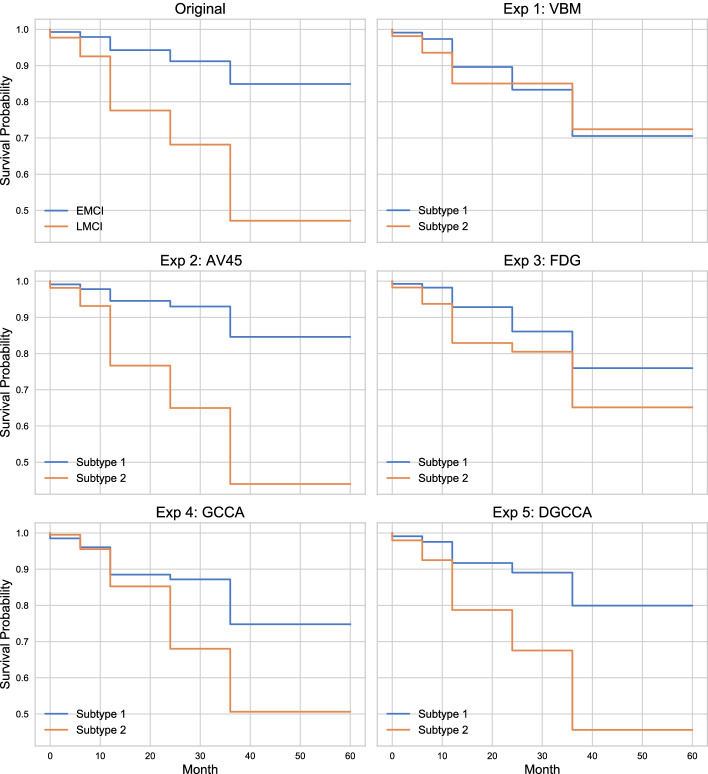
Conversion Curve the original MCI groups and each experiment from the Cox proportional hazard regression model, showing the probability of not converting to AD at the given month

**Fig. 8:**
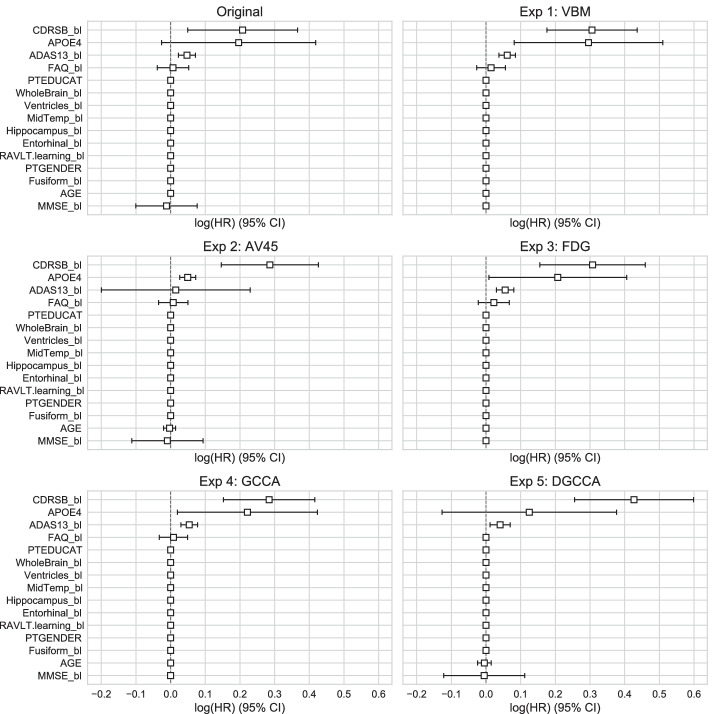
Log Hazard Ratio (HR) for the original MCI groups and each experiment from Cox proportional hazard regression model

To validate the identified MCI subtypes, we fitted Cox’s proportional hazard regression models for clusters generated by each experiment and the original MCI groups to investigate their conversion to AD. The model covariates include 5 cognitive assessments, 6 brain volume measures, and 4 data covariates. The conversion curves are shown in Fig. 7, and the log hazard ratio for all covariates are plotted in Fig. 8. The model fit and log rank test results are shown in Table 2. From the log rank test result and the conversion curves, compared to the original MCI groups, Exp 2 using AV45 features and Exp 5 using DGCCA features show the most distinctive trends for the two subtypes. Note that only 257 out of 976 total observations are on or after month 24, so the majority of observations are before month 24. While GCCA shows distinctive trends for two subtypes in the conversion curve, the two subtypes are not distinct before month 24. Consistent with in cluster similarity, see Fig. 5, AV45 has the most discriminative features out of 3 imaging modalities. The log hazard ratio plot shows that CDRSB and ADAS13 have the highest coefficients in the Cox regression model, whereas brain volume measures were zeroed out.

**Table 2:** Survival Analysis Evaluation

	Concordance	$\chi ^2$ statistic	*p*-value	$-\log _2(p)$
DX	0.882	47.924	4.430e-12	37.716
Exp 1 VBM	0.918	12.013	5.283e-04	10.886
Exp 2 AV45	0.900	39.734	2.911e-10	31.678
Exp 3 FDG	0.905	24.390	7.869e-07	20.277
Exp 4 GCCA	0.940	0.253	6.150e-01	0.701
Exp 5 DGCCA	0.925	34.906	3.460e-09	28.107

The $\chi ^2$ test statistics are from log rank test comparing survival curves of two clusters

While the baseline measures for 5 cognitive assessment and 6 brain volume from ADNI QT-PAD are used in survival analysis, we also used the longitudinal measures to plot progression curves up to 5 years after baseline for imaging-driven subtypes and the original MCI groups, see “Additional File 1: Fig. S1”. Subtypes from DGCCA features have non-overlapping 95% confidence intervals for ADAS13, CDRSB, MMSE and FAQ, and display more distinct progression trends in hippocampus and ventricles volume compared to EMCI and LMCI groups.

### Genetic association analysis

**Fig. 9:**
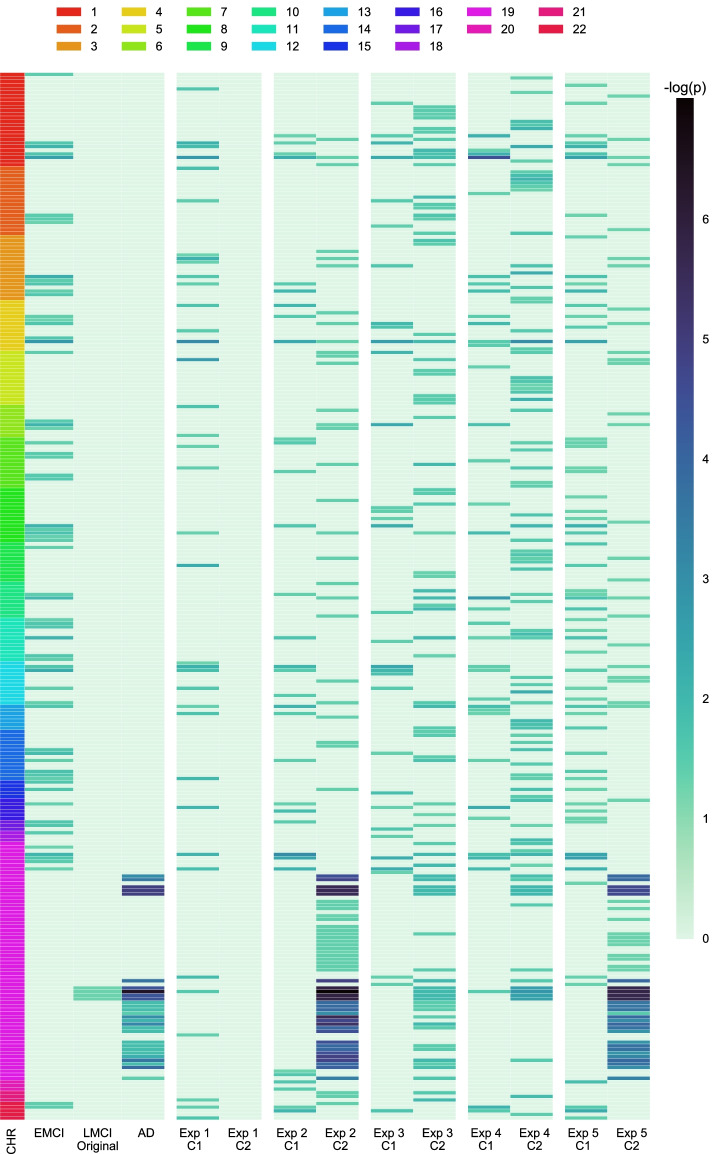
Heatmap of $-log_{10}(p)$ in genetic association analysis. After thresholding at $p=0.05$ with FDR correction, we include SNPs that are significant in at least one case control association test. SNPs are ordered by chromosomes, as shown on the left y-axis

Genetic association results from logistic regression for all experiments are summarized in Fig. 9, where the heatmap plots the $-\log _{10}(p)$ value for each SNP in different experiment. Only SNPs with at least one significant result amongst all subypes are shown in the heatmap. For visualization purpose, we ordered the SNPs by chromosome number from 1 to 22, marked by the color bar on the left. The full results with SNP, gene and *p*-value information are recorded in “Additional File 2: Table S1”.

The best known AD genetic risk SNPs from APOE, APOC1 and TOMM40 in chromosome 19 are identified by the AD group and all experiments except VBM in Exp 1. Note that the AD participants are not in experiment subtypes which are generated from MCI participants, and AV45 features in Exp 2 and DGCCA features from Exp 5 are able to identify many of the same signal as AD with even stronger signals (lower *p*-values), as shown in “Additional File 2: Table S1”.

There are also genetic markers identified by imaging experiment subtypes and not the original MCI. For instance, rs10961151 near the PRKCH gene is identified by Subtype 1 in Exp 5 using DGCCA feature, reported by a previous GWAS to be associated with paired helical filament (PHF) tau measurement [21]. On the other hand, rs149142 in the DACT1 and RPL9P5 gene is identified by Subtype 1 in Exp 5 and EMCI group, but not by single modality subtypes. This SNP was reported by a GWAS study [22] to be associated with cortical thickness.

Many genetic markers for AD are identified by imaging-driven MCI Subtype 2 using AV45 and DGCCA features with strong signals but not LMCI, which shows the limitation of the existing early vs late MCI groups. There are also genetic findings discovered by imaging-driven subtypes that were not found using the original MCI groups. This observation indicates the promise of these imaging-driven disease subtypes and their power in revealing interesting underlying genetic determinants. It warrants further investigation on the role of these subtype-derived genetic findings in disease status and AD progression in an independent cohort.

## Discussion

In this study, we have proposed to generate imaging-driven MCI subtypes from multimodal data using a fully unsupervised approach based on CCA. GCCA extends traditional CCA by applying transformation to more than 2 views of data, and is able to learn view-independent embeddings in addition to the view-dependent projections for each view. DGCCA can capture more variance in the original data with few features than its linear counterpart GCCA consistent with our previous work, and select both joint and unique features from its input views. We designed 5 experiments to compare features from single imaging modality and multiview methods. Two MCI subtypes were generated using unsupervised clustering where Subtype 1 has less disease severity and Subtype 2 has more. DGCCA and single modality features are able to generate valid subtypes that are distinct from the original MCI groups.

With this new grouping of participants, our validation studies demonstrate that DGCCA subtypes are discriminative in 6 brain volume measures (outperforming AV45), 5 cognitive assessment scores (outperforming VBM), AD conversion (outperforming VBM and FDG) and genetic markers (outperforming VBM and FDG). MCI subtypes generated from AV45 and DGCCA features can identify genetic markers in chromosome 19 with strong signals that AD group also identifies but the original MCI groups fail to. DGCCA subtypes show the best alignment with those from AV45 features, the most discriminative out of three modalities, although each modality is weighted the same in the DGCCA input. In the case that there are different views of data we want to learn from but don’t know how important they are, DGCCA can prove useful.

While subtypes from DGCCA features don’t outperform those from each single modality in every validation study, we show that DGCCA does effectively learn from all modalities. We also want to highlight that DGCCA reduces dimensions, where 20 features explain 68.85% variance of three modality of data, especially important given the limited sample size (n=308).

As to why DGCCA features don’t drastically improve upon single modalities, we can look at the test for cluster independence. When testing the assumption that the multimodal imaging data have a shared clustering assignment using the ROI features, this assumption holds only for AV45 and FDG. In other words, clusters from VBM features don’t align with those from AV45 and FDG using the original features. But this assumption holds for all pairs of the three modalities using DGCCA features. DGCCA is essentially learning from a subset of features from multiple modalities (as shown in Fig. 3 where some features are zeroed out) that have high correlation with each other. But these features might not necessarily correspond to those aligning with cluster assignments.

While we were able to show that the subtypes generated from DGCCA features are comparable to those generated from single modality and the original MCI groups, we are limited on the sample size and longitudinal observations. Our model uses simple feedforward networks on ROI level imaging features, but with DGCCA’s ability to learn non-linear transformation, the neural networks in DGCCA can be extended to more complex architectures to accommodate larger datasets and more complex features, e.g. convolutional layers for voxel-level imaging data and recurrent layers for genetic sequences. Some deep learning architectures can also help understand how the model learns from the input features and future work can expand on the interpretability of DGCCA.

## Conclusions

In this study, we show that DGCCA can learn low dimensional embeddings from multimodal neuroimaging data via non-linear transformations. Imaging-driven MCI subtypes generated from DGCCA embeddings align most with those generated from AV45 features, and show differential measures in brain volume, cognitive assessment, AD conversion and genetic markers. Our experiments demonstrate the potential of DGCCA in leveraging multimodal imaging data to learn disease structure beyond early and late MCI to faciliate early detection of AD.

## Methods

### Materials

Data used in the preparation of this article were obtained from the Alzheimer’s Disease Neuroimaging Initiative (ADNI) database (adni.loni.usc.edu) [23]. The ADNI was launched in 2003 as a public-private partnership, led by Principal Investigator Michael W. Weiner, MD. The primary goal of ADNI has been to test whether serial MRI, PET, other biological markers, and clinical and neuropsychological assessment can be combined to measure the progression of mild cognitive impairment (MCI, a prodromal stage of AD) and early AD. For up-to-date information, see www.adni-info.org.

#### Study participants

In this work, we analyzed 612 non-Hispanic Caucasian subjects with complete baseline measurements of 3 studied imaging modalities, genotyping data, cognitive assessments, brain volume measurements and visit-matched diagnostic information. Specifically, there are 219 controls (i.e., 154 healthy controls (HC) and 65 normal controls with significant memory concern (SMC)) and 393 cases (i.e., 195 patients with early MCI (EMCI), 113 patients with late MCI (LMCI), and 85 AD patients). Shown in Table 3 are their characteristics.

**Table 3:** Participant characteristics in our experiments at the baseline

Diagnosis	HC	SMC	EMCI	LMCI	AD	*p*-value
Number	154	65	195	113	85	-
Gender(M/F)	75/79	24/41	105/90	59/54	45/40	1.85E-01
Age(mean±sd)	75.44±6.40	71.97±5.25	71.04±7.04	72.66±8.53	73.96±8.30	6.69E-07
Education(mean±sd)	16.30±2.59	16.66±2.76	16.10±2.62	16.31±2.92	15.79±2.52	3.28E-01

There are totally 612 participants, where HC and SMC participants are grouped as controls (N$=$219), and EMCI and LMCI participants are grouped as cases (N$=$308). HC Healthy control; SMC Significant memory concern; EMCI Early mild cognitive impairment; LMCI Late mild cognitive impairment; AD Alzheimer’s disease. *p*-values were computed using one-way ANOVA (except for gender using $\chi ^2$ test)

#### Imaging data

The three imaging modalities used in this study are structural MRI [24] (sMRI, measuring brain morphometry), [$^{18}$F]florbetapir-PET [25] (AV45, measuring amyloid burden), and fluorodeoxyglucose -PET [26] (FDG, measuring glucose metabolism). The multi-modality imaging data were aligned to each participant’s same visit. The sMRI scans were processed with voxel-based morphometry (VBM) using the Statistical Parametric Mapping (SPM) software tool [27]. Generally, all scans were aligned to a T1-weighted template image, segmented into gray matter (GM), white matter (WM) and cerebrospinal fluid (CSF) maps, normalized to the standard Montreal Neurological Institute (MNI) space as 2$\times$2$\times$2 mm$^3$ voxels, and were smoothed with an 8mm FWHM kernel. The FDG-PET and AV45-PET scans were registered into the same MNI space by SPM and standard uptake value ratio (SUVR) was computed by intensity normalization based on a cerebellar crus reference region. The MarsBaR ROI toolbox [28] was used to group voxels into 116 regions-of-interest (ROIs). ROI-level measures were calculated by averaging all the voxel-level measures within each ROI. As mentioned above, participants in this work included 612 non-Hispanic Caucasian subjects with complete baseline ROI-level measurements of three modalities and visit-matched diagnostic information; see Table 3 for their characteristics.

#### Genetics data

Genotyping data were quality-controlled, imputed and combined as described in [29, 30]. Briefly, genotyping was performed on all ADNI participants following the manufacturer’s protocol using blood genomic DNA samples and Illumina GWAS arrays (610-Quad, OmniExpress, or HumanOmni2.5-4v1) [31]. Quality control was performed in PLINK v1.90 [32] using the following criteria: 1) call rate per marker $\ge 95\%$, 2) minor allele frequency (MAF) $\ge 5\%$, 3) Hardy Weinberg Equilibrium (HWE) test P $\le$ 1.0E-6, and 4) call rate per participant $\ge 95\%$. The resulting genotyping data include a total of 5,574,300 SNPs.

Given the large number of SNPs, we used GWAS catalog to select a subset [33] to be used in the genetic association analysis. Traits in the GWAS Catalog map to terms from the Experimental Factor Ontology (EFO) [34]. Searching for GWAS results using the trait “Alzheimer’s Disease” (EFO_0000249) and “Alzheimer’s disease biomarker measurement” (EFO_0006514), we obtained 1096 SNPs from 97 studies, and 2808 SNPs from 107 studies for each trait respectively. We then merged result for both traits with the ADNI genotyping data, obtaining 2,650 SNPs for all 612 participants.

#### Biomarker and clinical data

We used the ADNI data freeze for the Alzheimer’s Disease Modelling Challenge (QT-PAD) with longitudinal and heterogeneous measurements, see http://www.pi4cs.org/qt-pad-challenge. Along with 4 covariates (age, gender, APOE4 and education), we selected 11 AD-related baseline measures, including 5 cognitive assessment scores: Alzheimer’s Disease Assessment Scale 13-item cognitive subscale (ADAS13) [35], Clinical Dementia Rating Scale-Sum of Boxes (CDRSB) [36], Rey Auditory Verbal Learning Test score learning score (trial 5 score minus trial 1 score) [37], Mini-Mental State Examination (MMSE) [38], and Functional Assessment Questionnaire (FAQ) [39]; and 6 brain volume measures: whole brain, hippocampus, entorhinal, ventricles, middle temporal and fusiform.

### Multiview learning models

Given limited data and a rich feature space, a multiview learning method learns a single model from multiple input views data with reduced dimensions. We apply this method to learn a shared representation on three imaging modalities, sMRI (VBM), AV45 and FDG, where each modality corresponds to a single data view.

#### Generalized CCA (GCCA)

GCCA extends CCA [14] by learning correlated components from 2 data views by applying linear transformations. Given *J* views of data $X_j\in {\mathbb {R}}^{N\times p_j}$, where $X_j$ is the *j*-th view of the data, *N* is the number of data points, and $p_j$ is the number of features in view *j*, GCCA learns a view-dependent projection matrix $U_j\in {\mathbb {R}}^{p_j\times k}$ for each view, mapping to a view-independent shared representation or embedding $G\in {\mathbb {R}}^{N\times k}$ where *k* denotes the dimension of shared embedding space. The objective function of GCCA can be written as:

1 $$\begin{aligned} \underset{\begin{array}{c} U_1,U_2,\dots ,U_J, G \end{array}}{\text {minimize}} \; \sum _{j=1}^J \left\| G-X_j U_j\right\| _F^2 \quad \text {subject to} \; G^TG=I_k \end{aligned}$$

The optimal solution $\left( U_1^*,U_2^*,\dots ,U_J^*, G^*\right)$ and optimal objective value of problem (1) are as follows: $G^*$ contains the top *k* eigenvectors of $\sum _{j=1}^J X_j \left( X_j^T X_j\right) ^{-1} X_j^T$ as its columns, which we use as the share latent features,

2 $$\begin{aligned} U_j^* = \left( X_j^T X_j\right) ^{-1} X_j^T G^*, \; j=1,2,\dots ,J, \end{aligned}$$

and

3 $$\begin{aligned} \sum _{j=1}^J \left\| G^*-X_j U_j^*\right\| _F^2 = J k - \sum _{i=1}^k \lambda _i\left( \sum _{j=1}^J X_j \left( X_j^T X_j\right) ^{-1} X_j^T\right) , \end{aligned}$$

where $\lambda _i\left( \cdot \right)$ denotes the *i*-th largest eigenvalue of its matrix argument/input.

#### Deep generalized CCA (DGCCA)

**Fig. 10:**
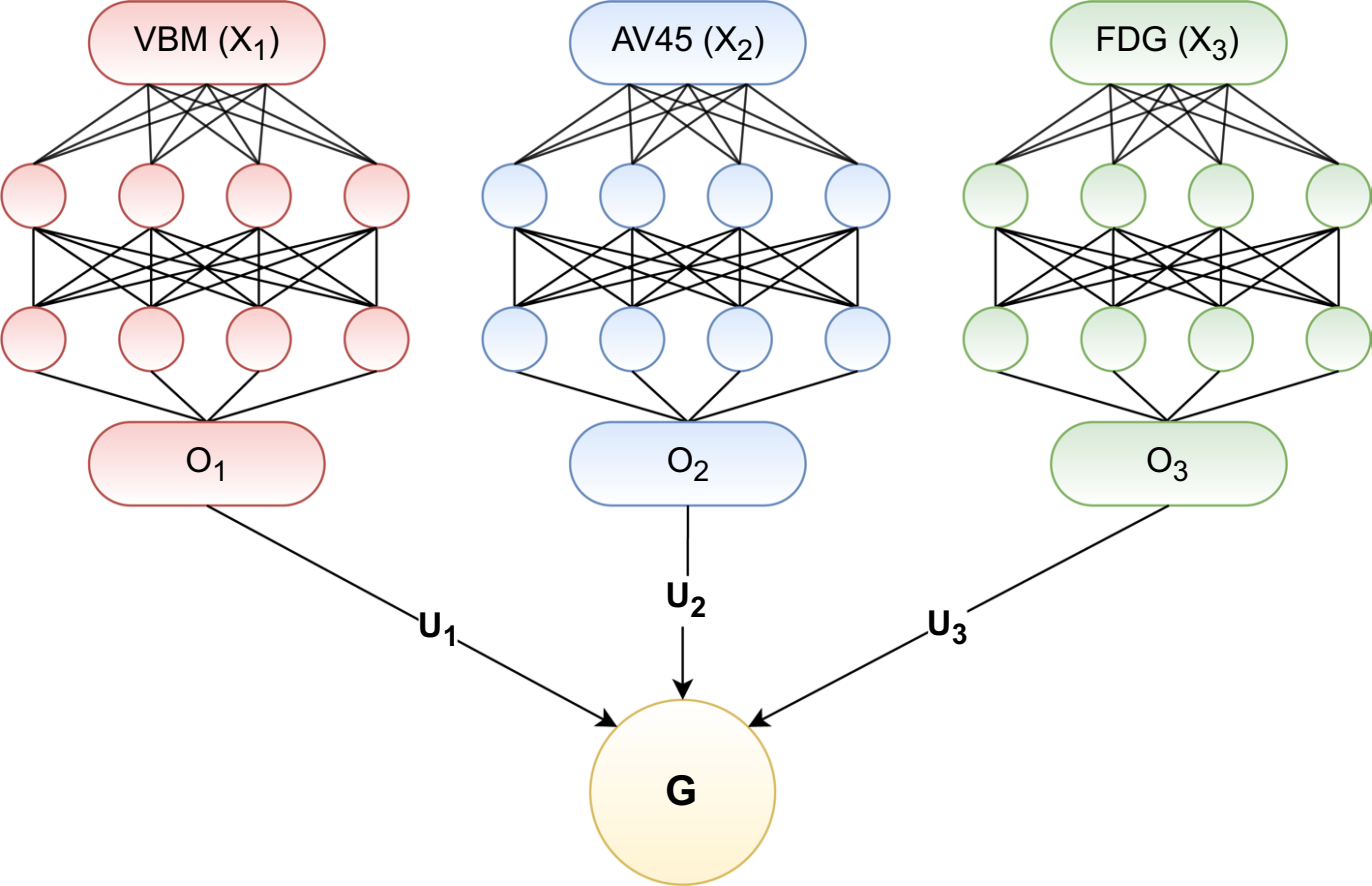
DGCCA architecture

DGCCA [17] extends GCCA further by introducing non-linearity by passing the each input view through its own neural network. The architecture of DGCCA is shown in Fig. 10. To learn the view-dependent projection matrices $\left( U_1^*, U_2^*, \dots , U_J^*\right)$ and view-independent embedding $G^*$, we minimize the objective function:

4 $$\begin{aligned} \begin{aligned} \left( \theta _1^*,\theta _2^*,\dots ,\theta _J^*\right) =&{{\,\mathrm{arg\,min}\,}}_{\left( \theta _1,\theta _2,\dots ,\theta _J\right) } \underset{\begin{array}{c} U_1,U_2,\dots ,U_J \\ G^TG=I_k \end{array}}{\text {minimize}} \; \sum _{j=1}^J \left\| G-O_j U_j\right\| _F^2 \\ \end{aligned} \end{aligned}$$

where $\theta _j$ and $O_j \in {\mathbb {R}}^{N\times q_j}$ are the vector of all network weights parameters and the output of the neural network for view *j*, respectively, and $U_j\in {\mathbb {R}}^{q_j\times k}$ and $G\in {\mathbb {R}}^{N\times k}$ are the view-dependent projection matrix for view *j* and the view-independent shared representation, respectively.

To train the neural networks, we need to compute the gradients of the DGCCA objective:

$$\begin{aligned} L\left( \theta _1,\theta _2,\dots ,\theta _J\right) = \sum _{i=1}^k \lambda _i\left( \sum _{j=1}^J O_j \left( O_j^T O_j\right) ^{-1} O_j^T\right) \end{aligned}$$

with respect to $\theta _1,\theta _2,\dots ,\theta _J$. This can be done by computing the gradients of $L\left( \theta _1,\theta _2,\dots ,\theta _J\right)$ with respect to $O_j$, and then backpropagation. As shown in [17], we have

5 $$\begin{aligned} \frac{\partial L\left( \theta _1,\theta _2,\dots ,\theta _J\right) }{\partial O_j} = 2 \left[ I_N-O_j \left( O_j^T O_j\right) ^{-1} O_j^T\right] G G^T O_j \left( O_j^T O_j\right) ^{-1}, \end{aligned}$$

where *G* has the top *k* eigenvectors of $\sum _{j=1}^J O_j \left( O_j^T O_j\right) ^{-1} O_j^T$ as its columns, and $I_N$ is the identity matrix of size $N \times N$.

#### Implementation

For GCCA, we used an existing implementation in Python [40], and extended this implementation for training the DGCCA model using the PyTorch library [41]. For both models, we used imaging data on 80% of MCI samples for training, and 20% for validation and tuning the model. The neural network for each input view consists of 2 hidden layers each of size 96 and with ReLU activation, a dropout layer where probability of zeroing out an element is 0.1 and the output layer has the same dimension as the input of 116 features. The networks were trained using the Adam optimizer with a learning rate of 0.0005 and weight decay of 0.01. To prevent overfitting, we also added an early stopping threshold with patience of 5, when the validation loss decreases by no more than $0.05\%$ of the max validation loss.

**Fig. 11:**
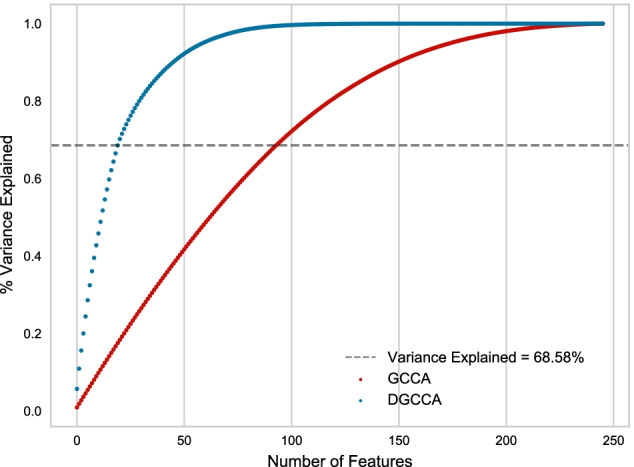
Variance Explained by components extracted from GCCA and DGCCA

When training DGCCA, *k*, the dimension of the shared embeddings *G* is a hyperparameter. We experimented with different values of *k* and found that embeddings with lower *k* can explain just as much variance of the original data as those from training with higher *k*, so we decided to set *k* at 20. To better compare GCCA and DGCCA, we then selected the top $k'$ features from embeddings *G* generated from both models that explain the same amount of variance of the original data. With $k=20$, $G_{dgcca}$ explains $68.58\%$ of variance, and using this threshold, we picked the top 94 features of $G_{gcca}$ which explain $68.66\%$ of variance. Consistent with our previous findings [13], DGCCA explains the same amount of variance in fewer latent features, see Fig. 11.

### Experimental design

In this section, we describe our experimental design on the features used and how the imaging-driven subtypes are generated.

#### Test for cluster independence

In our experiments, we would generate subtypes from multimodal data using unsupervised clustering, which makes the assumption that there is a shared clustering assignment of participants from all input views, in our case, the three imaging modalities. Before moving on to experiment design, we conducted a statistical test to check if this assumption holds [42], where the null hypothesis is that clusterings for two data views are independent of each other. In other words, if clusterings for two modalities are not independent of each other, we reject the null hypothesis, and the assumption that there’s a shared clustering on the given multimodal data holds.

Using the R package multiviewtest in [42], we conducted the test of cluster independence on both the 116 ROI features and 20 DGCCA embedding features ($O_j$) for comparison. Because this test can only be applied to two input views, we conducted it on three pairs of imaging modalities-VBM versus AV45, VBM versus FDG and AV45 versus FDG. This test was conducted for each of the 6 settings, and the resulting test statistics and *p*-value are recorded in Table 4. When we tested on the 116 ROI measures, we rejected the null hypothesis only for the pair FDG and AV45, indicating that there’s a shared clustering on FDG and AV45 data using the ROI measures, but we cannot concatenate VBM with either of these two modalities directly and generate valid subtypes. However, we rejected the null for all pairs of modalities when using 20 embeddings features generated from DGCCA, which means that we can apply clustering on the DGCCA embeddings learned from three modalities of data.

**Table 4:** Test independence of clusters generated from each pair of imaging modalities, using the original features ($X_j$) and DGCCA projected features ($X_jU_j$)

	Original features ($\textit{p}=116$)		DGCCA Projected Features ($k=20$)	
	Test Statistic	*p*-value	Test Statistic	*p*-value
VBM vs. AV45	1.089	0.99	**11.959**	<**0.0001**
VBM vs. FDG	1.742	0.38	**102.786**	<**0.0001**
AV45 vs. FDG	**12.522**	<**0.0001**	**11.381**	<**0.0001**

Significant results (*p*-value ≤ 0.05) are shown in bold

#### Experiments

**Fig. 12:**
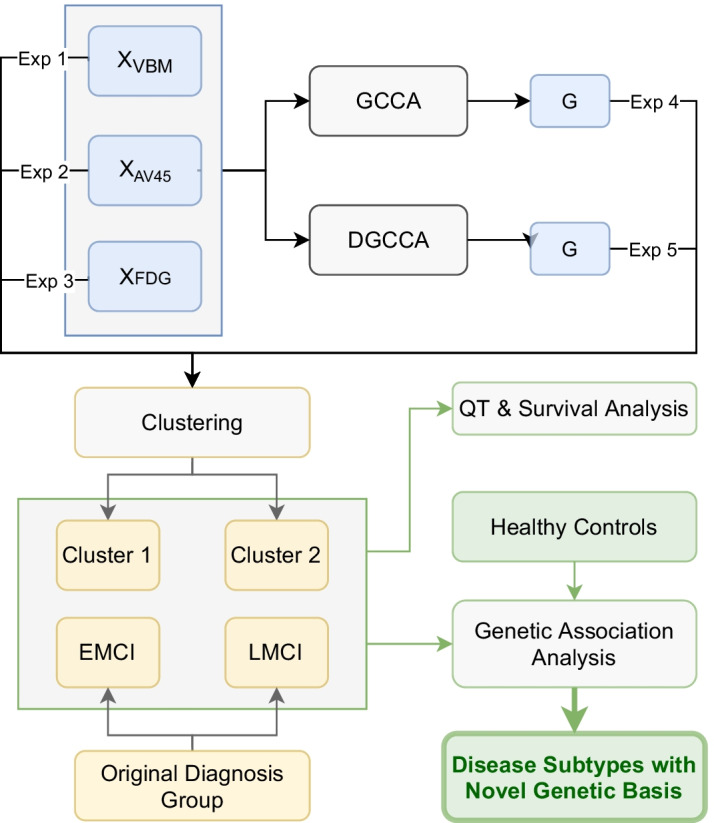
Experimental Flowchart

We designed a total of five experiments, and the flowchart is shown in Fig. 12. In addition to the shared embeddings *G* learned by GCCA and DGCCA, we also used the ROI features from each imaging modality for comparison. The number of features for the five experiments are recorded in Table 5, and these features are used in subsequent clustering to generate imaging-driven MCI subtypes.

**Table 5:** Comparison of features used in clustering

Experiments	Features Used for Clustering	# Features
Exp 1 VBM	$X_{\text {VBM}}$	116
Exp 2 AV45	$X_{\text {AV45}}$	116
Exp 3 FDG	$X_{\text {FDG}}$	116
Exp 4 GCCA	$G_{gcca}$	94
Exp 5 DGCCA	$G_{dgcca}$	20

The feature numbers used in Exps 5–6 were determined to explain $70\%$ of total data variance respectively; see also Fig. 11

**Fig. 13:**
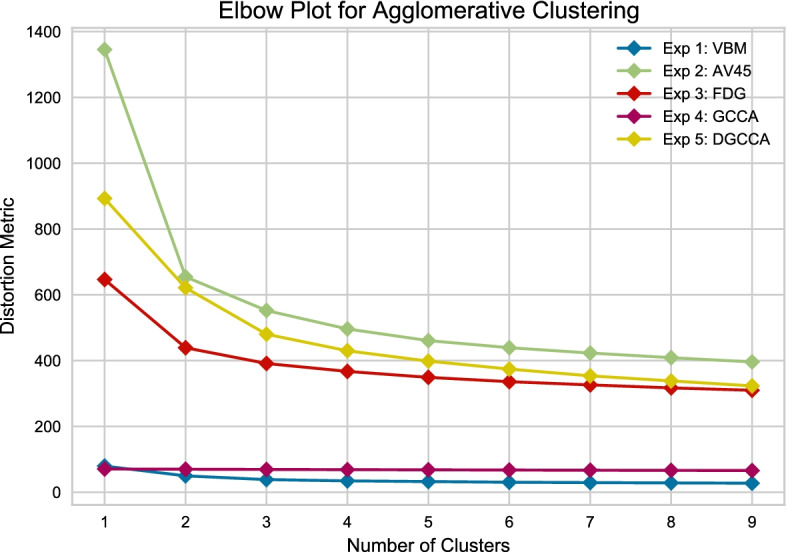
Elbow plot for picking cluster number using the distortion metric

We used agglomerative clustering to generate 2 clusters from 308 samples, similar to the original MCI group (EMCI and LMCI), and based on the elbow plot in Fig. 13. Subtype 1 is assigned to the cluster with higher EMCI to LMCI ratio, corresponding to less disease severity where Subtype 2 is of lower EMCI to LMCI ratio and more disease severity.

To evaluate the clustering result, we computed the Calinski-Harabasz (CH) score [43] and Silhouette score [44] as internal evaluation measures. CH score computes the ratio of the sum of between-cluster distances and the sum of within-cluster distances. Silhouette score measures how well a sample is matched to its own cluster versus the neighboring clusters. We also computed the Adjusted Mutual Information (AMI) [45] score to compare how similar the generated subtypes and the original EMCI/LMCI groups are.

### Validation studies

To validate our subtypes, we first conducted the Wilcoxon rank-sum test on each of the 5 cognitive assessment and 6 brain volume baseline measures from ADNI QT-PAD. To control for type 1 error, we adjusted the critical *p*-value of 0.01 using Bonferroni correction. We also conducted survival and genetic association analysis to further investigate the subtypes generated from each experiment.

#### Survival analysis

We conducted survival analysis by fitting a semi-parametric Cox’s proportional hazard model for each of the 5 experiments and original MCI groups for comparison. The Cox model (see Eq. 6) expresses the hazard function $h(t|X_i)$ at time *t* for individual *i*, given *p* covariates, denoted by $X_i$. The population baseline hazard $h_0(t)$ may change over time.

6 $$\begin{aligned} h(t|X_i) = h_0(t) \exp {\sum _{j=1}^{p} \beta _j X_{ij}} \end{aligned}$$

We first selected observations from participants from QT-PAD whose diagnosis at each visit is either MCI, AD or MCI converted to AD. We fitted the model on the resulting 976 observations from 304 MCI participants, with the 5 cognitive assessment 6 brain volume baseline measures and 4 covariates (age, gender, education and APOE e4) as model covariates, and added L1 and L2 penalty with their ratio being 1:1 to encourage sparse coefficients. Conversion curve is plotted for each subtype, showing the probability of “survival” (not converting to AD) at different time points, measured in months. The model fit is evaluated using concordance index, and the log rank test is also conducted to check the difference between the two subtypes/groups in each experiment.

In addition to survival analysis, we also plotted the progression curves using 5 cognitive assessments and 6 brain volume longitudinal measures, from month 0 to 60, to visualize the difference between the two subtypes in each experiment and the original MCI groups.

#### Genetic association analysis

Using the genetic data (2650 SNPs) on all 612 participants including controls (see Table 3), we ran PLINK case control association analysis [46] for each individual subtype generated in each experiment against the control group ($N=219$). As a comparison, we also conducted the case control analysis for the original group: EMCI vs. Control, LMCI vs. Control and AD vs. Control. While AD patients are not included in the MCI subtypes, we want to see if there’s overlap in terms of the identified genetic markers. The association analysis was ran using logistic regression given case control group as the phenotype, and with age, gender and education as covariates. APOE e4 allele (i.e., rs429358) was not included as a covariate in the analysis to check if our subtypes can identify the APOE e4 allele. We set the *p*-value threshold to 0.05 and controlled the false discovery rate (FDR) using the Benjamini-Hochberg (BH) procedure. Of note, given our modest sample size and SNPs pool, we selected a relatively liberal *p*-value threshold in order to suggest promising top findings for future investigation.

## Supplementary information

**Additional file 1: Fig. S1.**Progression Curves. For the 11 cognitive and biomarker measurements, while we only usedthe baseline measure in our cluster and survival analysis, we also plotted their progression curve using the longitudinal measures for the subtypes and the original MCI groups. The line plots are generated by aggregatingparticipants in the same subtype, where the line is the mean at a given time point and the shading is the 95%confidence interval.

**Additional file 2: Table S1.**Results for genetic association analysis in Fig. 9 sorted by chromosome number.Thresholding at p= 0.05 with FDR correction, only SNPs that are significant in at least one case control associationtest are included and only p-values for significant results are recorded. Mapped gene(s) for each SNP are shown asthey are recorded in GWAS Catalog - SNPs in multiple genes are separated by comma, interactions are separated by”x”, and upstream anddownstream genes are separated by a hyphen for intergenic SNPs.

## Data Availability

Data used in the preparation of this article were obtained from the ADNI database (adni.loni.usc.edu). The ADNI was launched in 2003 as a public-private partnership, led by Principal Investigator Michael W. Weiner, MD. The primary goal of ADNI has been to test whether serial magnetic resonance imaging (MRI), positron emission tomography (PET), other biological markers, and clinical and neuropsychological assessment can be combined to measure the progression of MCI and early AD.

## Abbreviations

AD: Alzheimer’s disease
ADNI: Alzheimer’s disease neuroimaging initiative
SMC: Significant memory concern
EMCI: Early mild cognitive impairment
LMCI: Late mild cognitive impairment
MRI: Magnetic resonance imaging
PET: Positron emission tomography
CCA: Canonical correlation analysis
GCCA: Generalized canonical correlation analysis
DGCCA: Deep generalized canonical correlation analysis
ROI: Region of interest
VBM: Voxel-based morphometry
AV45: [$^{18}$F]florbetapir or $^{18}$F-AV-45
FDG: Fluorodeoxyglucose or $^{18}$F-FDG
