# Supplementary material for: Deep multiview learning to identify imaging-driven subtypes in mild cognitive impairment
Source: BMC Bioinformatics. 2022 Sep 29;23(Suppl 3):402. doi: 10.1186/s12859-022-04946-x (PMC9523890; doi:10.1186/s12859-022-04946-x)

Deep Multiview Learning to Identify Imaging-driven Subtypes in Mild Cognitive Impairment

Yixue Feng, Mansu Kim, Xiaohui Yao, Kefei Liu, Qi Long, Li Shen and for the Alzheimer's Disease Neuroimaging Initiative

Supplemental Figure 1: Progression Curves. For the 11 cognitive and biomarker measurements, while we only used the baseline measure in our cluster and survival analysis, we also plotted their progression curve using the longitudinal measures for the subtypes and the original MCI groups. The line plots are generated by aggregating participants in the same subtype, where the line is the mean at a given time point and the shading is the 95\% confidence interval.

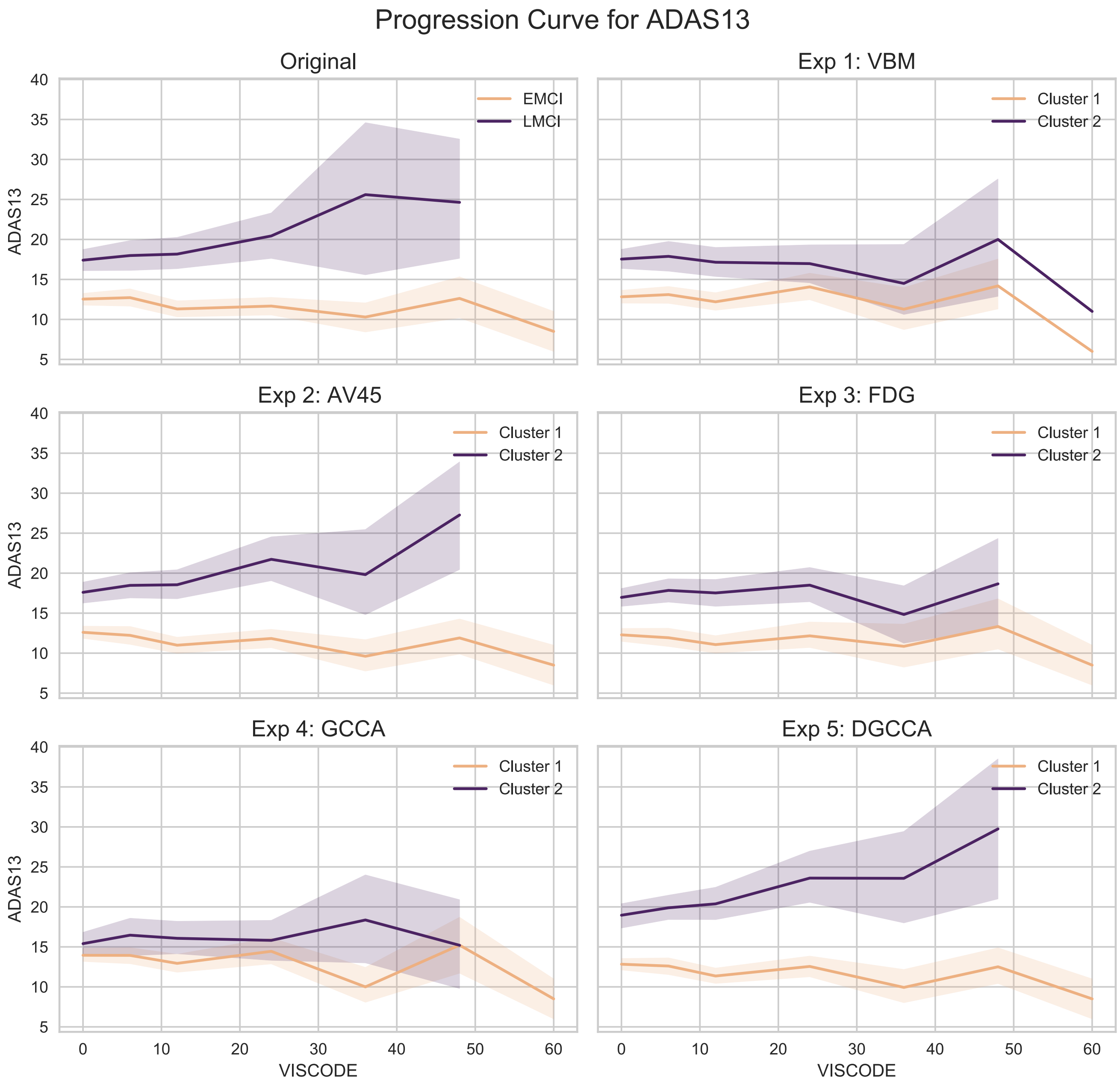

Supplement: Supplementary file 1 — Additional file 1: Fig. S1.Progression Curves. For the 11 cognitive and biomarker measurements, while we only usedthe baseline measure in our cluster and survival analysis, we also plotted their progression curve using the longitudinal measures for the subtypes and the original MCI groups. The line plots are generated by aggregatingparticipants in the same subtype, where the line is the mean at a given time point and the shading is the 95%confidence interval. [file 12859_2022_4946_MOESM1_ESM.pdf]
